# Sensor Proxy Mobile IPv6 (SPMIPv6)—A Novel Scheme for Mobility Supported IP-WSNs

**DOI:** 10.3390/s110201865

**Published:** 2011-02-01

**Authors:** Md. Motaharul Islam, Eui-Nam Huh

**Affiliations:** Department of Computer Engineering, College of Electronics and Information, Kyung Hee University, Yongin-si, 446-701, Korea; E-Mail: motahar@khu.ac.kr

**Keywords:** wireless sensor network, SPMIPv6, IP-WSN, IETF, 6LoWPAN, IEEE 802.15.4, IP sensor node

## Abstract

IP based Wireless Sensor Networks (IP-WSNs) are gaining importance for their broad range of applications in health-care, home automation, environmental monitoring, industrial control, vehicle telematics and agricultural monitoring. In all these applications, mobility in the sensor network with special attention to energy efficiency is a major issue to be addressed. Host-based mobility management protocols are not suitable for IP-WSNs because of their energy inefficiency, so network based mobility management protocols can be an alternative for the mobility supported IP-WSNs. In this paper we propose a network based mobility supported IP-WSN protocol called Sensor Proxy Mobile IPv6 (SPMIPv6). We present its architecture, message formats and also evaluate its performance considering signaling cost, mobility cost and energy consumption. Our analysis shows that with respect to the number of IP-WSN nodes, the proposed scheme reduces the signaling cost by 60% and 56%, as well as the mobility cost by 62% and 57%, compared to MIPv6 and PMIPv6, respectively. The simulation results also show that in terms of the number of hops, SPMIPv6 decreases the signaling cost by 56% and 53% as well as mobility cost by 60% and 67% as compared to MIPv6 and PMIPv6 respectively. It also indicates that proposed scheme reduces the level of energy consumption significantly.

## 1. Introduction

Recent advancement in micro-electro-mechanical and wireless communication systems have enabled the development of low cost, low power, multifunctional sensor nodes that are small in size and can communicate over short distances [1,2]. A sensor network is a special type of communication network that is composed of a large number of tiny sensor nodes that are densely deployed either inside the phenomenon or very close to it [1]. Sensors are generally equipped with data processing and communication capabilities. The sensing circuitry senses the environment surrounding the sensor and transforms them into an electric signal. The sensor sends such signal, usually via radio transmitter, to the sink node either directly or through other sensor nodes.

In the past, applications of sensor networks were thought to be very specific. The communication protocol of sensor network was also very simple and straightforward. Some researchers were even against the use of the internetworking concept in WSNs for reasons such as the resource constraints for layered architecture, the configuration problems of large numbers of devices, the essence of sensor nodes’ distinct identity, *etc.* But with the advent of the Internet of things and federated IP-WSNs, this demand is going to be blurred. The huge number of IPv6 addresses, the necessity for end to end communication and advancement of micro-electronics have changed the concept of the research community. Now a tiny sensor node can hold a compatible TCP/IP protocol stack [3], so we can now think of using the concept of internetworking protocols in IP-WSNs [4]. We can easily think of providing IPv6 address to individual sensor nodes since it provides around 6 × 10^23^ addresses per square meter of the Earth’s space.

The IPv6 over low power wireless personal area network (6LoWPAN) working group of the Internet Engineering Task Force (IETF) defines the manner in which IPv6 communication is to be carried out over IEEE 802.15.4 interface [5,6]. Although 6LoWPAN helps making the wide implementation of IP-WSN a reality and its end to end communication to the external world feasible, excessive signaling costs for sensor nodes because of too much tunneling through the air makes it difficult. Excessive signaling costs therefore become a barrier for the application of IP-WSNs especially in the case of the mobility scenario of individual sensor nodes or groups of nodes in different areas such as in a patient’s body sensor network, in industrial automation, *etc*. [7]. Nowadays most of the communication protocols are host based, which is practically infeasible for IP-WSNs since individual nodes need to participate in mobility related signaling. PMIPv6 which is a network based protocol, provides mobility support to any IPv6 host within a restricted and topologically localized portion of the network, without requiring the host to participate in any mobility-related signaling.

6LoWPAN based IP-WSNs may use the sensor network compatible PMIPv6 to introduce and to enhance the mobility scenario in a localized domain. With this thinking in mind, in this work we focus on the mobility in IP-WSNs with attention paid to an energy efficient network based communication protocol for IP-WSNs. In this regard, we have proposed the SPMIPv6 for IP-WSNs. In our proposed scheme we use sensor network-based localized mobility anchors, mobile access gateways, and multiple smart and fully functional sensor nodes which are IPv6 header stack-enabled. These all IP devices including the sensor nodes make it feasible to implement the SPMIPv6 protocol over IP-WSNs.

There are different application areas where IP-WSNs can be used such as industrial control, structural monitoring, healthcare, smart home, vehicle telematics, agricultural monitoring *etc*. [8]. In these scenarios, node to node communication is very important since these are the collaborative functions. In these cases IP-WSNs based on a mesh approach can enhance the communication scenario significantly, whereby individual sensor nodes can act as routers or fully functional devices, which is discussed in the route over routing issue in 6LoWPAN [8,9]. Moreover SPMIPv6 based IP-WSNs facilitate node to node seamless communication in different cases of the individual sensor node and sensor network mobility scenarios.

The main contributions of this paper are as follows:

- A novel IP-WSN architecture solely based on PMIPv6 and named SPMIPv6 is proposed for energy efficient mobility of individual sensor nodes or a group of sensor nodes. To the best of our knowledge this is the first paper reporting on a sensor network based localized mobility management protocol in an IP-WSN domain with special consideration to energy efficiency.
- We address the applicability of mobility issues of individual or group of IP sensor nodes in a patient care scenario.
- We propose functional architecture, and the respective message formats, sequence diagram, network model and evaluate the performance of the proposed protocol architecture.
- Mathematical analysis and simulation experiments are conducted to show the effectiveness of the proposed scheme. Our simulation results show that the proposed scheme effectively reduces the signaling cost and mobility cost in terms of the number of IP-WSNs and hops as compared to MIPv6 and PMIPv6, respectively. There is also a significant improvement in energy consumption for data payload and IP-WSN node density in the network.

The rest of the paper is organized as follows. Section 2 reviews background data related to PMIPv6 and 6LoWPAN. The proposed SPMIPv6 protocol architecture, along with its mobility scenarios, sequence diagram and message formats are presented in Section 3. Section 4 describes performance analysis and evaluation, Section 5 shows the experimental results, and finally Section 6 concludes the paper.

## 2. Background

### 2.1. Overview of PMIPv6

The foundation of PMIPv6 is based on MIPv6 in the sense that it extends MIPv6 signaling and reuses many concepts such as the home agent (HA) functionality [10,11]. However, PMIPv6 is designed to provide network-based mobility management support to a Mobile Node (MN) in a topologically localized domain [12]. Therefore, an MN is free from participation in any mobility-related signaling, and the proxy mobility agent in the serving network performs mobility-related signaling on behalf of the MN. Once an MN enters its PMIPv6 domain and performs access authentication, the serving network ensures that the MN is always on its home network and can obtain its home address on any access network. The serving network assigns a unique home network prefix to each MN, and conceptually this prefix always follows the MN wherever it moves within a PMIPv6 domain. From the perspective of the MN, the entire PMIPv6 domain appears as its home network. Accordingly, it is needless to configure the care of address at the MN. The new functional entities of PMIPv6 are the mobile access gateway (MAG) and local mobility anchor (LMA) [13]. The MAG typically runs on the access router (AR). The main role of the MAG is to detect the MN’s movements and initiate mobility-related signaling with the LMA on behalf of the MN. In addition, the MAG establishes a tunnel with the LMA for enabling the MN to use an address from it home network prefix and emulates the MN’s home network on the access network for each MN. On the other hand, the LMA is similar to the HA in MIPv6. However, it has additional capabilities required to support PMIPv6. The main role of the LMA is to maintain reachability to the MN’s address while it moves around within a PMIPv6 domain, and the LMA includes a binding cache entry for each currently registered MN. The binding cache entry maintained at the LMA is more extended than that of the HA in MIPv6 with some additional fields such as the MN identifier, the MN’s home network prefix, a flag indicating a proxy registration, and the interface identifier of the bidirectional tunnel between the LMA and MAG. Such information associates an MN with its serving MAG, and enables the relationship between the MAG and LMA to be maintained.

### 2.2. 6LoWPAN

The 6LoWPAN working group of the IETF has defined an adaptation layer for sending IPv6 packets over IEEE 802.15.4. The goal of 6LoWPAN is to reduce the sizes of IPv6 packets to make them fit in 127 byte IEEE 802.15.4 frames. The 6LoWPAN proposal consists of a header compression scheme, a fragmentation scheme, and a method for framing IPv6 link local addresses into IEEE 802.15.4 networks [5,6]. The proposal also specifies enhanced scalabilities and mobility of sensor networks. The challenge to 6LoWPAN lies in the sizable differences between an IPv6 network and an IEEE 802.15.4 network. The IPv6 network defines a maximum transmission unit as 1,280 bytes, whereas the IEEE 802.15.4 frame size is 127 octets. Therefore, the adaptation layer between the IP layer and the MAC layer must transport IPv6 packets over IEEE 802.15.4 links. The adaptation layer is responsible for fragmentation, reassembly, header compression and decompression, mesh routing, and addressing for packet delivery under the mesh topology. The 6LoWPAN protocol supports a scheme to compress the IPv6 header from 40 bytes to 2 bytes [7,14].

### 2.3. Related Work

In comparison to conventional WSNs, IP-WSN provides flexibility to access and manage individual sensor nodes locally or from remote sites through the Internet. This specific issue provides great significance to different mission specific applications. Our paper focuses on the energy efficient mobility of both IP sensor nodes and sensor networks which is based on PMIPv6 and 6LoWPAN. There are few approaches proposed in the literature regarding IP-WSNs and their mobility issues. RFC 5213 gives a complete description of PMIPv6 [11]. RFC 4944 depicts the overall aspects of 6LoWPAN, the packet format provided by IETF to ensure IPv6 communication over low-power, wireless personal area networks. In [9] the authors represent the overall scenario for extending IP to LoWPAN such as 6LoWPAN adaptation layer, header format encapsulation and compression and finally focus on addressing, routing, auto configuration and neighbor discovery issues. In [14], its authors propose a network mobility protocol to support 6LoWPAN, but they do not consider the 6LoWPAN node mobility. The respective authors only consider network mobility which is performed with the help of mobile routers as well as they also do not consider unique addresses for each sensor node. In [8] the authors describe some real life design and application spaces for 6LoWPANs which show the importance of 6LoWPANs in near future. In [15] the authors propose a dual addressing scheme for 6LoWPAN-based WSNs. It combines a global unicast address to cope with association link changes and node mobility, and it links local addresses to reduce the overhead. Although our proposed scheme considers global unicast address only, it is energy efficient because of using SPMIPv6 for communication protocol.

## 3. Proposed SPMIPv6 Protocol

### 3.1. SPMIPv6 Protocol Architecture

**Figure 1.**
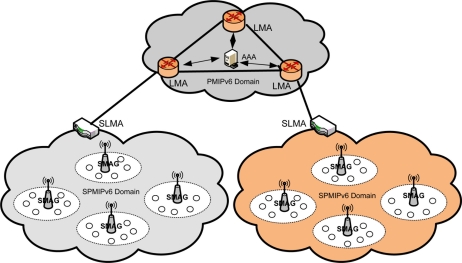
Sensor Proxy Mobile IPv6 Architecture.

To introduce energy efficient mobility we propose Sensor Proxy Mobile IPv6 (SPMIPv6) for IP-WSN. It is a localized mobility management protocol based on PMIPv6. The SPMIPv6 architecture consists of a sensor network-based localized mobility anchor (SLMA), a sensor network-based mobile access gateway (SMAG) and many fully functional IPv6 header stack enabled IP sensor nodes. We discuss the individual module of the proposed architecture with the help of Figure 1.

#### 3.1.1. SLMA

The SLMA acts as a topological anchor point for the entire SMAG and IP sensor nodes. The main role of the SLMA is to maintain accessibility to the sensor node while the node moves within or outside of the SPMIPv6 domain. The SLMA includes a binding cache entry for each sensor node, encapsulations and decapsulation section and SMAG information table. The binding cache entry at the SLMA is used for holding the information of the mobile sensor node. It includes different information such as a sensor node address, the sensor node’s home network prefix, and a flag bit indicating sensor proxy registration.

The SLMA has sufficient memory to hold all of these necessary records. It has sufficient power supply and processing capability. It also acts as the interfacing device between SPMIPv6 domain and PMIPv6 domain. As an interfacing entity, it facilitates the communication of the IP-WSN with the Internet world. In this scheme, authentication, authorization, and accounting (AAA) service has been integrated within SLMA. We name this integrated service as sensor network based authentication, authorization, and accounting (SAAA) schemes. The SAAA scheme helps the SMAG and sensor node to obtain secured mobility in the SPMIPv6 domain. It also facilitates authentication services for each fully functional sensor nodes.

#### 3.1.2. SMAG

The SMAG acts like a sink node in a traditional sensor network. In SPMIPv6 it acts like an access gateway router with a main function of detecting sensor node movement and initiating mobility-related signaling with the sensor node’s SLMA on behalf of the sensor node. It can move with its member sensor node as a small IP-WSN domain similar to the body sensor network of a patient. It consists of different functional module such as routing, neighbor discovery, sensor information table, adaptation module and interfacing module to the sensor node and SLMA. Routing module performs the efficient data transmission among individual sensor node and facilitates the end to end communication. Neighbor discovery module performs neighbor discovery and duplicate address detection functionality. Adaptation module performs the task of transmitting IPv6 packet over IEEE 802.15.4 link as mentioned in 6LoWPAN adaptation layer. The sensor information table provides the up to date information of sensor nodes to the SLMA. It works with the close association of the binding cache entry of the SLMA, and the two interfacing modules communicate with the SLMA and the sensor nodes.

#### 3.1.3. IP-Sensor Node

SPMIPv6 domain consists of numerous sensor nodes based on IPv6 addresses. We consider the domain as a federated IP sensor domain. There are two types of sensor node. One type contains the tiny TCP/IP communication protocol stack with adaptation layer and IEEE 802.15.4 interface. This type can forward information to other node of similar type as well as information sensing from the environment. Actually this type of sensor node acts as a mini sensor router. The other type of sensor node has the protocol stack and environment sensing capability but can forward the sensed information to nearby mini sensor router node. However, both of these types are able to perform end to end communication.

### 3.2. Operational Architecture of SPMIPv6

**Figure 2.**
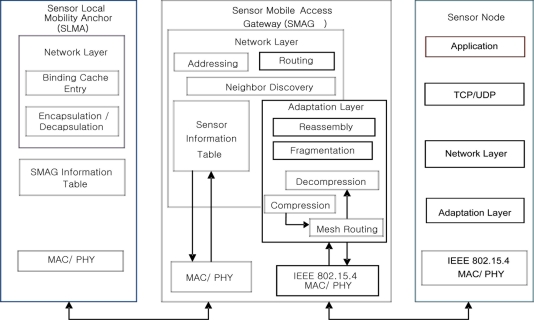
Operational Architecture of SPMIPv6.

Figure 2 shows the detail operational architecture of the SPMIPv6 which includes SLMA, SMAG and IP sensor nodes. And it shows how these entities communicate each other by different types of interfaces. The SMAG needs two or more interfaces for the communication with different access network such as SPMIPv6 based IP-WSN and external PMIPv6 network. It includes the network layer, adaptation layer and physical layer functionality. The network layer provides different functionality such as addressing, routing, neighbor discovery and the data structure for holding IP sensor node information. The most important layer is adaptation that ensures mesh routing, compression and decompression, and fragmentation and reassembly. The physical layer provides access to different physical interfaces. SLMA holds network related information such as binding cache entry, encapsulation and decapsulation. Binding cache entry provides data structure to hold different information such as new SPMIPv6 flag, link local address for each interface, home prefix, bi-directional tunnel interface identifier, access technology and time stamp. Finally, the IP sensor node works as a very smart tiny 6LoWPAN based node. It holds a tiny TCP/IP protocol stack. Moreover it holds the basic functionality of adaptation layer. All the sensor nodes consist of IPv6 addresses for local and global communications.

### 3.3. Application Scenarios of IP-WSN based on SPMIPv6

This section includes different SPMIPv6 based IP-WSN applications scenarios [8] briefly. A complete description is beyond the scope of this paper.

#### 3.3.1. Industrial Monitoring

SPMIPv6 based IP-WSN applications for industrial monitoring can be related with a broad range of methods to increase productivity, energy efficiency, and safety of industrial operations in engineering facilities and manufacturing plants. Many industries currently use time-consuming and expensive manual monitoring to predict failures and to schedule maintenance or replacements in order to avoid costly manufacturing downtime. A SPMIPv6 based IP-WSN could be inexpensively installed and provide more frequent and more reliable data. The deployment of SPMIPv6 based IP-WSNs can reduce equipment downtime and eliminate manual equipment monitoring that is costly to be carried out. Additionally, data analysis functionality can be placed into the network, eliminating the need for manual data transfer and analysis.

#### 3.3.2. Structural Monitoring

Intelligent monitoring in facility management can make safety checks and periodic monitoring of the architecture status highly efficient. Powered nodes can be included in the design phase of a construction or battery-equipped nodes can be added afterwards. All nodes are static and manually deployed. Some data such as normal room temperature is not critical for security protection, but event-driven emergency data must be handled in very critical manner.

#### 3.3.3. Healthcare

SPMIPv6 based IP-WSNs are envisioned to be heavily used in healthcare environments. Although hospital scenarios can be handled differently, IP-WSN provides great potential to ease the development of new services by getting rid of cumbersome wires and simplifying patient care in hospitals and for home care as the World is rapidly graying. The worldwide population of elderly people over age 65 is expected to be more than double from 357 million to 761 million by 2025 [16]. The speed with which this age-structural change is taking place implies an urgent need for solutions that will relieve the mounting pressure on our health-care systems as well as support a better quality of life and quality of care for our aged.

#### 3.3.4. Connected Home

The connected home or smart home is with no doubt an area where SPMIPv6 based IP-WSN can be used to support an increasing number of services: home safety/security, home automation and control, personal healthcare, smart appliances and home entertainment systems. In home environments SPMIPv6 based IP-WSN networks typically comprise a few dozen units and probably in the near future a few hundreds of nodes of various natures: IP sensors, actuators and connected objects.

#### 3.3.5. Vehicle Telematics

SPMIPv6 based IP-WSN play an important role in intelligent transportation systems. Incorporated in roads, vehicles, and traffic signals, they contribute to the improvement of safety of transporting systems. Through traffic or air-quality monitoring, they increase the possibilities in terms of traffic flow optimization and help reducing road congestion.

#### 3.3.6. Agricultural Monitoring

Accurate temporal and spatial monitoring can significantly increase agricultural productivity. Due to natural limitations, such as a farmers’ inability to check the crop at all times of day or inadequate measurement tools, luck often plays too large a role in the success of harvests. Using a SPMIPv6 based IP-WSN, indicators such as temperature, humidity, soil condition, can be automatically monitored without labor intensive field measurements. For example, SPMIPv6 based IP-WSN could provide precise information about crops in real time, enabling businesses to reduce water, energy, and pesticide usage and enhancing environment protection. The sensing data can be used to find optimal environments for the plants. In addition, the data on the planting condition can be saved by sensor tags, which can be used in supply chain management.

### 3.4. Mobility Scenario of IP-WSN Based on SPMIPv6

**Figure 3.**
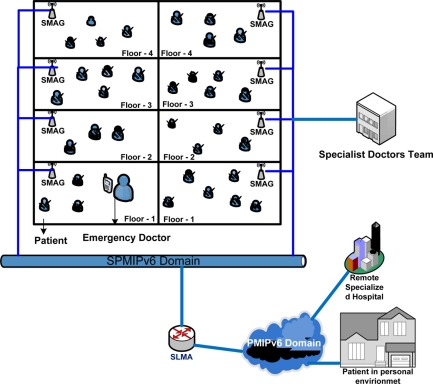
Mobility scenario in SPMIPv6-based patient care unit.

To represent mobility scenarios, we consider a state-of-the-art technology based patient care unit in a specialized hospital. Figure 3 represent such type of scenario where a patient can get special care both in the hospital and personal home care and from a remote specialized hospital. In this scenario, we try to depict different type of mobility in SPMIPv6 environment [17]. Sensor nodes are deployed on the body of the patients as well as all over the environment. The patient care unit ensures real time care and observation of the patients from a central specialized doctor’s forum. In case of emergency, patient can move from one place to another with its complete set-up so that seamless connectivity with the sophisticated medical equipment remains established and doctor can monitor the patient online from the central doctor’s research group. All the floors are considered as single SPMIPv6 domain. Inside the SPMIPv6 domain there are six floors considered as individual SMAG domain.

The mobility issue considered in this scenario are: Case-I: Movement of nodes within the same SMAG domain of the SPMIPv6 domain, Case-II: Movement of nodes between different SMAGs of the same SPMIPv6 domain, Case-III: Movement of nodes between different SMAGs of different SPMIPv6 domains, Case-IV: Movement of a SMAG-based personal area network (PAN) within the same SPMIPv6 domain, Case-V: Movement of a SMAG-based PAN between different SPMIPv6 domains, Case-VI: Patient monitoring in personal home environment. These scenarios are explained below:

Case-I: In this case, the mobility of the nodes will be handled by the appropriate SMAG, without the involvement of the SLMA. This, the simplest mobility scenario, arises frequently in hospital management: a patient can move within the PAN of a single branch of the hospital for different purposes such as exercise and getting fresh air.
Case-II: In this case, mobility will be handled by the appropriate SMAG with minimal initiative from the SLMA. The initial coordination will be performed by the SLMA alone; then the SMAG will oversee the remaining procedures. In our hospital management model, a patient can move from one PAN to another PAN in the same branch of the hospital.
Case-III: In this case, mobility is inter-domain, using the public PMIPv6 domain. The LMA, AAA, and SLMA will coordinate with one other. In our hospital management model, a patient can move on an emergency basis from one PAN of a hospital branch to a PAN of another branch of the same hospital.
Case-IV: In this case, mobility is based on the network mobility (NEMO) protocol [14], confined to the same domain. Only the SLMA and corresponding SMAGs will be involved. In our hospital management model, a patient with the whole set up can move from one PAN to another PAN.
Case-V: This case is also based on NEMO protocol, but is much different from Case-IV. In our hospital management model, a patient can move on an emergency basis with its whole setup from one branch of a hospital to the more specialized branch of the same hospital.
Case-VI: Due to the increasing number of aging demographic group, we consider this case so that a patient can be monitor continuously from the patient’s personal environment as discussed in our recent paper.

Although some of the mobility scenarios may seem complicated in case of IP based wireless sensor network, still they have been considered for the future research. Because of the essence of huge deployment of IP sensor network in health care, departmental store and industrial automation, this mobility scenario will eventually come true in the near future.

### 3.5. Message Flow in SPMIPv6

In the SPMIPv6 architecture authentication features are integrated in SLMA instead of performing these two functions separately. Proxy binding update message and SAAA query message are combined together and named as binding update and authentication query. As well as SAAA reply and proxy binding acknowledge message are merged together and named as binding acknowledge and authentication reply. For this reason the number of communications is greatly reduced in SPMIPv6 with comparison to conventional PMIPv6. The reduced communication scenario has been drawn and compared with the conventional PMIPv6 sequence diagram. The steps of the sequence diagram in both PMIPv6 and SPMIPv6 are discussed below.

**Figure 4.**
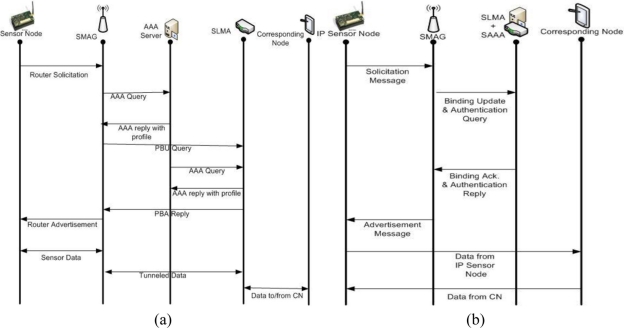
**(a)** Sequence diagram in PMIPv6. **(b)** Sequence diagram in SPMIPv6.

Figure 4(a) depicts the sequence diagram of the message flow with respect to PMIPv6. For the simplicity in Figure 4(a), we combine more than one communication within single step.

Step 1: When a sensor node first attaches to a SMAG domain, the access authentication procedure is performed using the sensor node address.
Step 2: After successful access authentication, the SMAG obtains the sensor node’s profile from the AAA service policy store. This profile contains the sensor node’s address, SLMA address, supported address configuration mode, and other associated information.
Step 3: The SMAG sends a proxy binding update (PBU) message, including the MN address, to the sensor node’s SLMA on behalf of the sensor node.
Step 4: Once the SLMA receives the PBU message, it checks the policy store to ensure that the sender is authorized to send the PBU message. If the sender is a trusted SMAG, the SLMA accepts the PBU message.
Step 5: The SLMA sends a proxy binding acknowledgment (PBA) message, including the MN’s home network prefix option, and establishes a route for the sensor node’s home network prefix over the tunnel to the SMAG.

Figure 4(b) shows the sequence diagram of the overall message flow with respect to SPMIPv6. The number of communications is noticeably reduced in SPMIPv6 with comparison to conventional PMIPv6 shown in Figure 4(a).

Step 1: When the location of an IP sensor node is changed, it sends solicitation message for the nearest SMAG discovery mechanism.
Step 2: SMAG sends binding update and authentication query to SLMA integrated with SAAA.
Step 3: In response to the binding update and authentication query message, SLMA integrated with SAAA send back binding acknowledge and authentication reply message.
Step 4: Finally SMAG send the advertisement message to respective IP sensor node. And thus the IP sensor node is connected to the nearest SMAG.
Step 5: Now the sensor node is able to communicate with the corresponding node based on SPMIPv6. Thus data can be transmitted from IP sensor node to correspondence node and *vice versa*.

### 3.6. Proxy Binding Message Format of SPMIPv6

**Figure 5.**
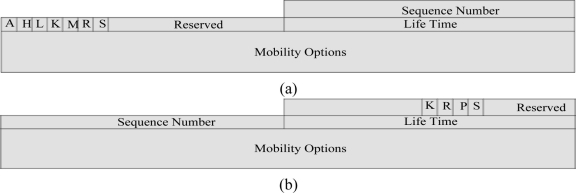
**(a)** SPMIPv6 PBU Message Format. **(b)** SPMIPv6 PBA Message Format.

In the proposed proxy binding update (PBU) and proxy binding acknowledgement (PBA) messages in Figure 5, we have specified a flag bit S. If this S flag is set, it indicates SMIPv6 operations. If the S bit is not set, this indicates non-SPMIPv6 operations. The meanings and descriptions of the other flags are described in different request for comments (RFC) [11,18–20]. The mobility options header plays an important role for the different types of mobility scenario. Some of the mobility header options are hand off indicators options, access technology type options, link layer identifier options, link local address options and time stamp options.

### 3.7. Gateway Router Solicitation and Advertisement Message Format of SPMIPv6

**Figure 6.**
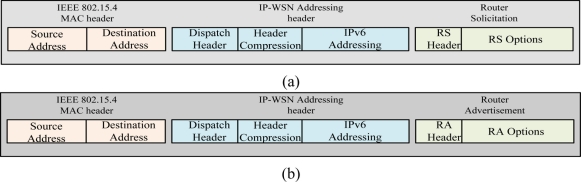
**(a)** SPMIPv6 Router Solicitation (RS) Message Format. **(b)** SPMIPv6 Router Advertisement (RA) Message Format.

Figure 6 depicts router solicitation (RS) and router advertisement (RA) message format of SPMIPv6. Figure 6 (a) shows the RS message which is sent by IP-WSN sensor node. The IEEE 802.15.4 MAC header’s source and destinations address set to the source and destination sensor node’s MAC address. Dispatch of IP-WSN addressing header indicates the compressed IPv6 header. Header compression section compresses different fields of IP-WSN addressing header. Router solicitation message’s RS options enable IP-WSN gateway to obtain the sensor node’s MAC address, link-local address and sensor node’s ID. The RA message format has two options such as home network prefix (HNP) and address options. The HNP options are used to emulate IP-WSN sensor node’s home network.

## 4. Performance Analysis and Evaluation

For analyzing signaling costs, we use a two-dimensional (2D) random walk model [21–23] based on the properties of regular, absorbing Markov chains. Random walk mobility models are designed for dynamic location areas and are suitable for modeling user movement when mobility is generally confined to a limited geographical area. Such scenarios include homes, vehicles, hospitals, and department stores.

### 4.1. Network Mobility Model

**Figure 7.**
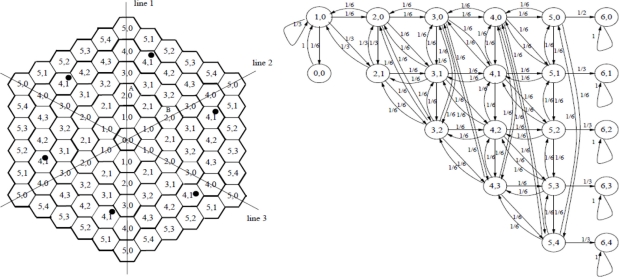
Type Classification and State diagram of a six-sublayer PAN area model.

Mobility of sensor nodes and the network is the major advantage of IP-WSNs over conventional static wireless sensor networks. In this paper, mobility is the key concern in the design and performance analysis of IP-WSNs. The mobility model plays a key role in studying different mobility management strategies such as registration, handoff, and authentication *etc.* A mobility model with minimum assumptions and simple to analyze is very useful for an IP-WSN. Most of wireless network performance studies assume that the coverage areas are configured as a hexagonal or square shaped. We assume that IP-WSN networks to be configured with hexagonal topology. Sensor nodes for an IP-WSN area are assumed to have identical movement patterns within and across IP-WSN. A 2D hexagonal random walk mobility model can be used to study the movement pattern of the movable sensor nodes. In this paper, we will use a network model subject to some modification for the six-layer personal area network model, with n = 6. In our network model, an IP-WSN consists of a cluster of hexagonal sensor nodes as shown in Figure 7 [21].

The SMAG at the center of the IP-WSN area is sublayer 0. The six-subarea clusters are shown in Figure 7; lines 1–3 divide the cluster into six equal pieces. Exchange of any two pieces has no effect on the structure of the cluster. Sensor nodes in cells of the same type will leave the cells with the same routing pattern. A sensor node can move to any one of its six neighbors with a uniform probability of 1/6. Each sensor node is denoted by <x, y>, where x indicates that the SMAG is in subarea x, and y is one of the types of subarea x. States <5, 0>, <5, 1>, <5, 2>, <5, 3> and <5, 4> are in the boundary of the IP-WSN and therefore called the boundary states. The state transition diagram of the regular Markov chain corresponding to the random walk model for the six-layer IP-WSN area is shown in Figure 7. Movement into any boundary state indicates inter-IP-WSN mobility, which can be used to study binding update costs.

### 4.2. Analysis of Signaling Costs

The state transition diagram in Figure 7 shows that there are no transient sets in the model, but only a single ergodic set with only one cyclic class. Hence, the properties of regular Markov chains can be exploited to analyze the behavior of the proposed model [22].

Let P be the regular transition probability matrix; then the steady state probability vector π can be solved using the following equations:

$$\pi P=\pi \;\;\text{and }\;\sum_{i=1}^{m}\pi_{i}=1$$

Here, m is the number of states and P is the fundamental matrix for the regular Markov chain. This is given by:

$$Z=\left[Z_{\mathrm{ij}}\right]={\left(I-P+A\right)}^{-1}$$

where

1. *A* is a limiting matrix determined by *P*, and the powers of *P^n^* approach the probability matrix *A*;
2. Each row of *A* consists of the same probability vector π = {π_1_,π_2_,.....π_*n*_}, *i.e.*, *A* = ξπ, where ξ is the column vector with all entries equal to 1; and
3. *I* is the identity matrix.

The matrix *Z* can be used to study the behavior of the regular Markov chain, and, using this matrix, one can compute the mean number of times that the process is in a particular state.

Let *Y_j_*(*k*) is the number of times that a process is in state *S_j_* in the first *k* steps; then *M_i_*[*y*(*k*)], the mean number of times the process is in state *S_j_* starting from state *S_i_*, is given by:

$$M_{i}\left[y_{j}{}^{\left(k\right)}\right]\to \left(Z_{\mathrm{ij}}-\pi_{i}\right)+k\pi_{j}$$

The total number of boundary updates in *k* steps, starting from state *S_i_*, can be computed from the total number of times that the process is in the boundary states starting from state *S*_*j*_, the initial state. The average number of location updates (*U*_*bu*_) in the analytical model, is given by:

$$U_{\mathrm{bu}}=M_{i}\left[y_{1}{}^{\left(k\right)}\right]+M_{i}\left[y_{2}{}^{\left(k\right)}\right]+M_{i}\left[y_{3}{}^{\left(k\right)}\right]+M_{i}\left[y_{4}{}^{\left(k\right)}\right]$$

$$\text{So}:\;U_{\mathrm{bu}}=\sum_{n=1}^{4}M_{i}\left[y_{n}{}^{\left(k\right)}\right]$$

where 1, 2, 3 and 4 are the boundary states in the model:

Generalizing:

$$U_{\mathrm{bu}}=\sum_{n=1}^{\alpha }M_{i}\left[y_{n}{}^{\left(k\right)}\right]$$

We can use the above equation to determine the number of binding update messages. Because we need to send a binding update message whenever the sensor node moves between IP-WSNs, each time a node enters a boundary state, a binding update message is generated. Therefore, we need to determine the expected number of times that the process enters into a boundary state within k steps.

Thus, sensor nodes need to send *U_bu_* binding update messages, given that the sensor node experiences a total of k transitions between SMAGs. Therefore, the ratio of intra-IP-WSN mobility is denoted as *M*_int*ra_IP_WSN*_ and is expressed by:

$$M_{\text{int}\mathrm{ra}\_\mathrm{IP}-\mathrm{WSN}}=\left(k-U_{\mathrm{bu}}\right)/k$$

The ratio of inter-IP-WSN mobility is denoted as *M*_int*er_IP_WSN*_ and is expressed by:

$$M_{\text{int}\mathrm{er}\_\mathrm{IP}-\mathrm{WSN}}=U_{\mathrm{bu}}/k$$

### 4.3. Signaling Cost Analysis of IP-WSN

We have evaluated our proposed model based on signaling cost, mobility cost and energy consumption. In the subsequent section we discuss the signaling cost analysis and energy consumption analysis with the help of different parameters mentioned in Table 1. Mobility cost is evaluated based on signaling cost. To evaluate total signaling costs, we compare the results of our analytical model with those of MIPv6 and PMIPv6.

**Figure 8.**
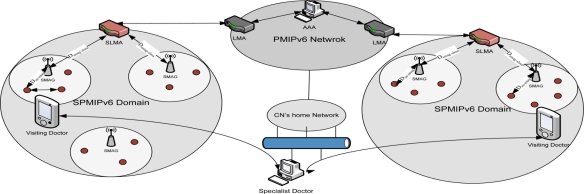
Analytical model for the performances analysis of SPMIPv6.

Figure 8 depicts the analytical model for the performance analysis of the proposed model. It consists of two different SPMIPv6 domains which are connected over the PMIPv6 based inter network. This Figure is the analytical representation of Figure 1. We may consider the individual SPMIPv6 domains as the different floors of the patient care unit. Distance between SMAG and IP sensor node is denoted by D_sn-smag_ and the distance between SMAG and SLMA is denoted by D_smag-slma._ The corresponding node (CN) considered as the specialist doctor is communicating with the visiting doctor. There are some visiting doctors who take the patient in continuous basis. In this analytical model, different distances are used for calculating signaling cost. The cost is incurred due to transmission of data and control signal. The cost will vary dew to different types of signal transmission. There may be five types of mobility scenario which has already been discussed in the earlier section. If patients move to a remote site then the signal will be transmitted through PMIPv6 domain. Other wise signal transmission will be restricted with in the SPMIPv6 domain.

From Equation (1) the total signaling cost (SC_mipv6_) of the proposed scheme based on MIPv6 is calculated by summing up the individual cost of intra-IP-WSN mobility (M_intra-ip-wsn_. C_sd_) and inter-IP-WSN mobility (M_inter-ip-wsn_.(C_sd_ + C_bu_)). Where, C_sd and_ C_bu_ are the sensor mobility cost and binding update cost respectively:

(1) $${\text{SC}}_{\text{mipv6}}=M_{\text{intra-ip-wsn}\text{. }}C_{\text{sd}}+M_{\text{inter-ip-wsn}.}\left(C_{\text{sd}}+C_{\text{bu}}\right)$$

where C_sd_ and C_bu_ are calculated in term of MIPv6 as follows:

$$\begin{array}{c} C_{\text{sd}}=\alpha .\left({\text{RS}}_{\text{mipv6}}+{\text{RA}}_{\text{mipv6}}\right)D_{\text{sn-smag}}\\ C_{\text{bu}}=\alpha .\left({\text{BU}}_{\text{mipv6}}+{\text{BA}}_{\text{mipv6}}\right)D_{\text{sn-smag}}+\beta .\left({\text{BU}}_{\text{mipv6}}+{\text{BA}}_{\text{mivp6}}\right)D_{\text{smag-slma}} \end{array}$$

From Equation (2) the total signaling cost (SC_pmipv6_) of the proposed scheme based on PMIPv6 is calculated by summing up the individual cost of intra-IP-WSN mobility (M_intra-ip-wsn_. C_sd_) and inter-IP-WSN mobility (M_inter-ip-wsn_.(C_sd_ + C_bu_)). Where, C_sd and_ C_bu_ are the sensor mobility cost and binding update cost respectively:

(2) $${\text{SC}}_{\text{pmipv6}}=M_{\text{intra-ip-wsn }\cdot }C_{\text{sd}}+M_{\text{inter-ip-wsn}\cdot }\left(C_{\text{sd}}+C_{\text{bu}}\right)$$

where C_sd_ and C_bu_ are calculated in term of PMIPv6 as follows:

$$\begin{array}{c} C_{\text{sd}}=\alpha .\left({\text{RS}}_{\text{pmipv}6}+{\text{RA}}_{\text{pmipv}6}\right)D_{\text{sn-smag}}\\ C_{\text{bu}}=\beta .\left({\text{PBU}}_{\text{pmipv}6}+{\text{PBA}}_{\text{pmipv}6}\right)D_{\text{smag-slma}} \end{array}$$

From Equation (3) the total signaling cost (SC_spmipv6_) of the proposed scheme based on SPMIPv6 is calculated by summing up the individual cost of intra-IP-WSN mobility (M_intra-ip-wsn_. C_sd_) and inter-IP-WSN mobility (M_inter-ip-wsn_.(C_sd_ + C_bu_)). Where, C_sd and_ C_bu_ are the sensor mobility cost and binding update cost respectively:

(3) $${\text{SC}}_{\text{spmipv6}}=M_{\text{intra-ip-wsn}\;\cdot \;}C_{\text{sd}}+M_{\text{inter-ip-wsn }\cdot }\left(C_{\text{sd}}+C_{\text{bu}}\right)$$

where C_sd_ and C_bu_ are calculated in term of SPMIPv6 as follows:

$$\begin{array}{c} C_{\text{sd}}=\alpha .({\text{RS}}_{\text{spmipv6}}+{\text{RA}}_{\text{spmipv6}})\;D_{\text{sn-smag}}\\ C_{\text{bu}}=\beta {\text{.(PBU}}_{\text{spmipv6}}{+\text{PBA}}_{\text{spmipv6}}\text{) }D_{\text{smag-slma}} \end{array}$$

### 4.4. Energy Consumption analysis of IP-WSN

For energy consumption analysis, we consider an IP-WSN with densely deployed IP sensing devices. The network consists of two types of IP sensing device: fully functional IP sensing devices (IP-FFD) and reduced functional IP sensing devices (IP-RFD). IP-FFD holds the complete 6LoWPAN protocol stack and performs the routing functions. IP-RFD performs the sensing functions and forwards the data to the IP-FFD. We have followed the energy consumption model used in [2,24–27]. Table 2 represents the used parameter values in the energy consumption model. Here, E^tx^ and E^rx^ is the distance-independent amount of energy consumed by the transmitter and receiver electronics and the digital processing of each. Here α and β are path loss exponent (2 < α < 5) and a constant [J/bit m^2^], r is a transmission range. L_ctrl_ is the length of control packets in bits, L_E_ is the energy needed by the transmitter device to transmit or receive a packet, and T is the time period between two consecutive topological changes of the IP-WSN. *nffd*_*p*_(*d_i_*) indicates the number of fully functional neighboring node for a path p and range d:

$$E=E^{\mathrm{tx}}+\beta *d^{\alpha }+E^{\mathrm{rx}}$$

Since same type of transmitting and receiving device is concerned in IP-WSN:

$$\begin{array}{l} E^{\mathrm{tx}}=E^{\mathrm{rx}}=E_{\mathrm{dec}}\\ E=2*E_{\mathrm{dec}}+\beta *d^{\alpha } \end{array}$$

$$\begin{array}{c} C_{i}^{\mathrm{ctrl}}(r)=\left[L_{\mathrm{ctrl}}*\beta *r^{\alpha }+\left(n_{i}(r)+1\right)*L_{\mathrm{ctrl}}*L_{E}\right]\frac{1}{T}\\ C_{i}^{\text{inf}}(p)=\left[\sum_{i=1,\;j=2}^{N}\left({\mathrm{nffd}}_{p}\left(d_{i}\right)+1\right)*L_{\mathrm{data}}*\beta d_{i,\;j}{}^{\alpha }+\left(n_{p}\left(d_{i}\right)+1\right)*L_{\mathrm{data}}\right]*L_{E}\\ C_{i}^{\mathrm{total}}(p)=\sum_{i=1}^{N}\left[C_{i}^{\mathrm{ctrl}}(r)\right]+C_{i}^{\text{inf}}(p) \end{array}$$

So energy consumed by MIPv6 scheme can be calculated by the following mathematical derivation. Here $E_{i}^{\mathrm{mipv}6}$ indicates energy consumption by the MIPv6 scheme:

$$E_{i}^{\mathrm{mipv}6}=SC_{i}^{\mathrm{mipv}6}*C_{i}^{\mathrm{total}}(p)$$

Energy consumed by PMIPv6 scheme can be calculated by the following mathematical derivation. Here $E_{i}^{\mathrm{pmipv}6}$ indicates energy consumption by PMIPv6 scheme:

$$E_{i}^{\mathrm{pmipv}6}=SC_{i}^{\mathrm{pmipv}6}*C_{i}^{\mathrm{total}}(p)$$

Finally energy consumed by SPMIPv6 scheme can be calculated in the same way. Here $E_{i}^{\mathrm{spmipv}6}$ indicates energy consumption by SPMIPv6 scheme:

$$E_{i}^{\mathrm{spmipv}6}=SC_{i}^{\mathrm{spmipv}6}*C_{i}^{\mathrm{total}}(p)$$

## 5. Experimental Results

In this section, we present the results of experiments evaluating the performance of our scheme, and compare the performance of our proposed scheme to MIPv6 and PMIPv6. First, we evaluate the performance of our proposed approach by mathematical analysis. Then, we set signaling cost and mobility related cost for the number of IP based sensor nodes and number of hops traverse during the mobility phase in order to evaluate the consequences of our proposed scheme with PMIPv6 and MIPv6. Finally, we summarize the key characteristics of our proposed approach as compared to PMIPv6 & MIPv6 approach.

We have implemented the model and evaluate the parameter such as the signaling cost and mobility related cost as presented in this paper. The experiments were conducted on a computer with AMD Athlon™ 2.5 GHz CPU and 2 GB primary memory. We use network simulator tool NS2 on VMware based Linux Fedora 4.

**Figure 9.**
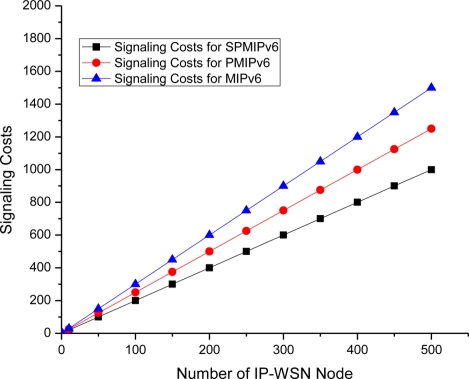
Number of IP-WSN nodes *vs.* Signaling Cost.

The Figure 9 depicts the signaling cost with respect to the number of IP-WSN node in term of the MIPv6, PMIPv6 and SPMIPv6. Signaling cost increases as the number of IP-WSN nodes increases. However, SPMIPv6 incurs much less signaling cost compared to MIPv6 and PMIPv6 as the number of IP-WSN nodes increases. The SPMIPv6 scheme reduces signaling cost by 60% and 56% with respect to MIPv6 and PMIPv6 in terms of the number of IP-WSN nodes.

**Figure 10.**
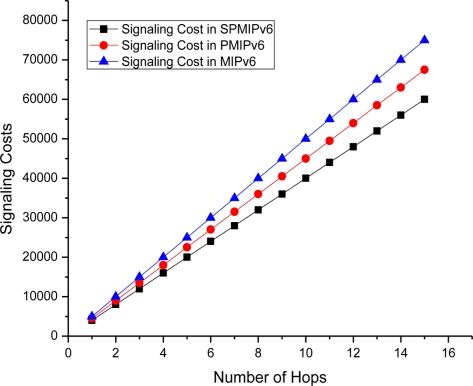
Number of Hops *vs.* Signaling Cost.

Figure 10 depicts the signaling cost with respect to the numbers of hops the data or signal traverses to reach the destination node. In this case we consider a maximum 15 hops. Signaling cost increases linearly as the number of hops increases. The proposed scheme shows better performance with respect to both PMIPv6 and MIPv6. In all cases signaling cost increases in a linear pattern. SPMIPv6 scheme reduces signaling cost by 56% and 53% with respect to MIPv6 and PMIPv6 in terms of the number of hops.

**Figure 11.**
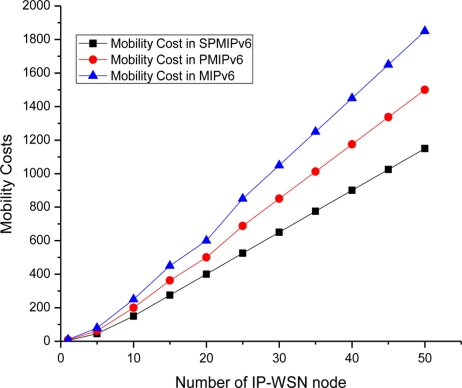
Number of IP-WSN nodes *vs.* Mobility Cost.

Figure 11 shows the mobility cost with respect to the number of mobile sensor nodes in both MIPv6 PMIPv6 and SPMIPv6. Mobility cost increases as the number of mobile sensor node increases. SPMIPv6 enjoy less mobility cost with respect to MIPv6 and PMIPv6.

Difference of mobility cost becomes more reasonable when the number of mobile sensor node increases. The SPMIPv6 scheme reduces signaling cost by 62% and 57% with respect to MIPv6 and PMIPv6 in terms of the number of IP-WSN nodes.

**Figure 12.**
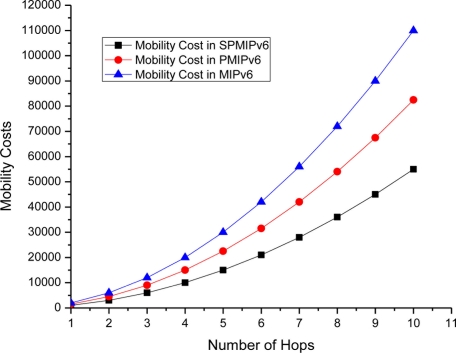
Number of Hops *vs.* Mobility Cost.

Figure 12 shows the mobility cost with respect to number of hops used by the mobile sensor nodes in MIPv6, PMIPv6 and SPMIPv6. Mobility cost increases exponentially as the number of hops increases. In this scenario also, our proposed scheme (SPMIPv6) enjoys less mobility cost with respect to MIPv6 and PMIPv6. Proposed SPMIPv6 scheme reduces 67% and 60% mobility cost with respect to MIPv6 and PMIPv6 in terms of the number of hops.

**Figure 13.**
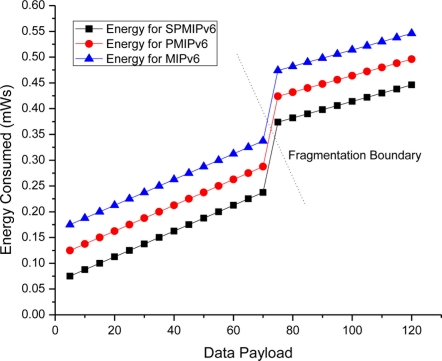
Data payload *vs.* Energy consumption.

The Figure 13 shows the energy consumption with respect to payload. Energy consumption is linear in MIPv6, PMIPv6 and SPMIPv6. However, due to fragmentation overhead, energy consumption increased rapidly and then it represents the linear characteristics again.

**Figure 14.**
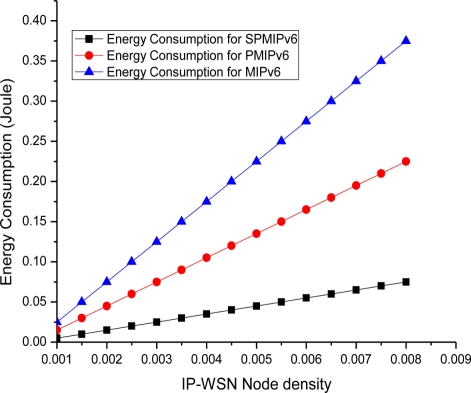
Node Density *vs.* Energy Consumptions.

The Figure 14 depicts the energy consumption with respect to the IP-WSN node density in terms of the MIPv6, PMIPv6 and SPMIPv6. Energy consumption increases as the IP-WSN node density increases. Our proposed scheme increases the performance linearly compared to MIPv6 and PMIPv6. Therefore, the energy consumption increases more rapidly as the density of IP-WSN node increases.

## 6. Conclusions

In this paper we present a sensor proxy mobile IPv6 protocol that enhances the mobility issue in IP-WSNs. Mobility in sensor networks is the most challenging issue that must be addressed with special attention to energy efficiency. Since SPMIPv6 is a sensor network-based localized mobility management protocol, it meets the demands for energy efficiency in terms of reducing signaling costs and mobility costs. Here we also present its architecture and messages formats and evaluate its performance by analyzing signaling costs and mobility costs. Our experiments show that SPMIPv6 reduces both signaling cost and mobility cost in comparison to MIPv6 and PMIPv6. In this paper, we focus on a 6LoWPAN based IP-WSN of the same vendor and protocol stack. In future we will focus on sensor networks consisting of multi vendors and heterogeneous protocol stacks.

## Figures and Tables

**Table 1.** System Parameters.

**Symbol**	**Description**
**BU**	Binding Update Message
**BA**	Binding Acknowledgement Message
**PBU**	Proxy Binding Update Message
**PBA**	Proxy Binding Acknowledge Message
**D_smag_slma_**	Distance between SMAG and SLMA
**D_sn_smag_**	Distance between SN and SMAG
**α**	Unit transmission cost in a wireless link
**β**	Unit transmission cost in a wired link
**RS**	Router Solicitation Message
**RA**	Router Advertisement Message
**C_sd_**	Sensor Mobility Cost
**C_bu_**	Binding Update Cost

**Table 2.** Parameter values.

**Parameter**	**Value**
**No of IP-WSN Node (N)**	25∼120
**Network Area (A)**	120 × 120 M
**Node density (ρ)**	0.00173∼0.00833
**Initial Energy**	2 J
**Transmit/Receive electronics (L_E_)**	50 nJ bit^−1^ m^2^
**Transmission Power**	5.85 × 10^−5^ W
**Number of SMAG**	1–10
**Transmission range (r)**	25 m
**Packet size**	2 KB
